# 

**Published:** 1781-09

**Authors:** 


### PROMOTION.

*Auguſt* 28.—Mr. Thomas Merrick to be ſurgeon to the 96th regiment of foot, in the room of Mr. Wood.

### DEATHS.

### DEATHS.

*July* 24. at Netherſole houſe, near Canterbury, aged 72 years, John Wincheſter, Eſq; Member of the Corporation of Surgeons, London. He was formerly an eminent ſurgeon in Norfolk-ſtreet in the Strand, but declined practice ſeveral years ago on a conſiderable eſtate being left to him for life by —— Marſh, Eſq; in conſequence of his ſetting the leg of a favourite dog, being before a ſtranger to him. His eſtate (about 1500 l. *per annum*) now deſcends to Mr. Marſh's nephew. He has left one ſon, a captain in the army, and a daughter married to Sir Edward Dering, Bart.——*Auguſt* 18. Suddenly, at an inn at Maidenhead, Berkſhire, Mr. Hawkins, apothecary, of New Palace Yard, Weſtminſter. ——*Sept.* 2. At Port Glaſgow, in Scotland, Mr. John M'Colme, ſurgeon to the weſtern fencible regiment.——17. At Brompton, near Chatham, Kent, Mr. Hodſkin, ſurgeon and apothecary, formerly a navy ſurgeon.

SECTION

### SECTION IV.

### MONTHLY CATALOGUE.

1. REPORTS of the Humane Society, instituted in the year 1774, for the recovery of perfons apparently drowned. For the years 1779 and 1780. 8vo. *Rivington*, London, 1781. 157 pages.

2. Differtatio inauguralis de cinerea cerebri fubftantia. Auctore *Chriftiano Frederico Ludwig*. 4to. Lipfiæ, 1779. 34 pages, *with a copper plate*.

3. Rapport fur plufieurs queftions propofées à la Société Royale de Medecine, par M. l'Ambaffadeur de la Religion, de la part de S. A. Eminentiffime Monfeigneur le Grand Maitre, &c. lû dans la féance de la Société Royale de Medecine, tenüe au Louvre le 5 Decembre, 1780. *i. e.* Anfwers to feveral queftions propofed to the Royal Medical Society (at Paris) by the Maltefe Ambaffador, at the requeft of his Eminency the Grand Mafter, &c. read at a meeting of the Royal Medical Society, held at the Louvre, December 5, 1780. 4to. Malta, 1781. 54 pages.

The ifland of Malta, founded on a rock, has only a fingle burial place, which is in the vaults of a church that was overthrown by an earthquake quake in January 1780. The College of Phyſi-cians at Malta recommended the ſtopping up the vaults, and adviſed againſt rebuilding on the ruins of the church. The queſtions propoſed therefore to the Royal Society at Paris were, 1. Whether it was right to ſtop up the vaults, and whether the church may be rebuilt on the ſame ſpot? 2. After what length of time may the vaults be opened again with ſafety? 3. What precautions are neceſſary to prevent the infection which the digging the earth in this ſpot might occaſion, and when will the danger of ſuch in-fection be over? 4. What are the objections to the practice of burying in churches? To the firſt of theſe queſtions the Society anſwer, That it was prudent to ſtop up the vaults. In reply to the ſecond they obſerve, that the ſmall pox was lately epidemic at Malta; that the plague prevailed there in 1676, and that a part of the vaults in queſtion was ſet apart for the bodies of thoſe who died of the latter diſeaſe. They re-mark, that the time contagious miaſmata pre-ſerve their activity is uncertain; but they think it would be prudent to ſuffer twenty-four or twenty-five years more to elapſe before the earth is opened in theſe vaults. There will then have been a ſpace of 128 or 129 years ſince the time of of the plague, and the deſtruction of the dead bodies will be complete; on this occaſion the Society quote the example of the magiſtrates of Marſeilles in a recent inſtance. In 1720, when the plague raged in that city, the dead bodies were interred in the burial ground belonging to the Celeſtines. A propoſal was lately made to build on that ſpot, but the magiſtrates very properly oppoſed it. In anſwer to the third queſtion the Society mention all the precautions that will be requiſite in opening the vaults; and in their reply to the fourth they offer a variety of arguments to prove the impropriety of burying the dead in churches or even within the walls of great towns.

4. *Henrick Palmaz Leweling*, D. A. D. &c. Anatomiſche erklaerung der original figuren von Andreas Veſalius; *i. e.* An anatomical deſcription of the original plates of Andrew Veſalius. By *Henry Palmaz Leweling*, M. D. profeſſor of anatomy and ſurgery in the univerſity of Ingolſtadt. Vol. I. Folio. Ingolſtadt, 1780. p. 120.

The whole of this work is to be comprized in ſeven volumes. This firſt volume contains the Oſteology.

